# X-ray diffraction using focused-ion-beam-prepared single crystals

**DOI:** 10.1107/S1600576720003143

**Published:** 2020-04-14

**Authors:** Tina Weigel, Claudia Funke, Matthias Zschornak, Thomas Behm, Hartmut Stöcker, Tilmann Leisegang, Dirk C. Meyer

**Affiliations:** a Institute of Experimental Physics, Technische Universität Bergakademie Freiberg, Leipziger Strasse 23, 09596 Freiberg, Germany; b Samara State Technical University, Molodogvardeyskaya Street 224, 443100 Samara, Russian Federation

**Keywords:** X-ray diffraction, sample preparation, focused ion beams

## Abstract

High-quality single-crystal X-ray diffraction measurements are a prerequisite for obtaining precise and reliable structure data and electron densities. The single crystal should therefore fulfill several conditions, of which a regular defined shape is of particularly high importance for compounds consisting of heavy elements with high X-ray absorption coefficients. The absorption of X-rays passing through a 50 µm-thick LiNbO_3_ crystal can reduce the transmission of Mo *K*α radiation by several tens of percent, which makes an absorption correction of the reflection intensities necessary. In order to reduce ambiguities concerning the shape of a crystal, used for the necessary absorption correction, a method for preparation of regularly shaped single crystals out of large samples is presented and evaluated. This method utilizes a focused ion beam to cut crystals with defined size and shape reproducibly and carefully without splintering. For evaluation, a single-crystal X-ray diffraction study using a laboratory diffractometer is presented, comparing differently prepared LiNbO_3_ crystals originating from the same macroscopic crystal plate. Results of the data reduction, structure refinement and electron density reconstruction indicate qualitatively similar values for all prepared crystals. Thus, the different preparation techniques have a smaller impact than expected. However, the atomic coordinates, electron densities and atomic charges are supposed to be more reliable since the focused-ion-beam-prepared crystal exhibits the smallest extinction influences. This preparation technique is especially recommended for susceptible samples, for cases where a minimal invasive preparation procedure is needed, and for the preparation of crystals from specific areas, complex material architectures and materials that cannot be prepared with common methods (breaking or grinding).

## Abstract 2

This study demonstrates a new preparation method for single-crystal X-ray diffraction samples using a focused ion beam. The results of the structure determination and electron density maps with differently prepared samples are discussed, to evaluate this new method.

## 1. Introduction   

Single-crystal X-ray diffraction (SC-XRD) is a widely used analysis method for structure determination. Besides structural information, in particular the electron density (ED) distribution is experimentally available (Massa, 2011 ▸). For the reconstruction of high-quality ED data, measurements up to high diffraction angles are necessary (Wölfel, 1987 ▸). Extinction and absorption effects of the crystal as well as ambient conditions, *e.g.* density of air, cooling water temperature or generator stability (Weigel *et al.*, 2015 ▸), may have significant influences on the SC-XRD data quality. To allow correction of the reflection intensities for the influence of absorption, the crystals should have a regular and simple-to-identify shape. Irregularly shaped crystals with many facets generate a wider distribution of different X-ray beam path lengths. Wrong or not correctly determined facets can then distort the data set when an absorption correction is applied. This leads to errors mainly in the atomic coordinates and the ED. For more regularly shaped crystals, errors mainly occur in the dis­place­ment parameters (Massa, 2011 ▸).

Commonly, small crystals with irregular shape, as grown or as cut from larger crystals, are used for SC-XRD (Hsu *et al.*, 1997 ▸). Regularly shaped crystals are usually prepared by grinding of single-domain crystals into spheres (Massa, 2011 ▸; Abrahams, Reddy & Bernstein, 1966 ▸; Iyi *et al.*, 1992 ▸; Etschmann & Ishizawa, 2001 ▸; Abrahams & Marsh, 1986 ▸). Alternatively, a focused-ion-beam (FIB) preparation, which allows nano-fabrication of samples with defined size and shape, could be utilized. This method of sample preparation is known from the field of electron microscopy (Reyntjens & Puers, 2001 ▸; Jarmar *et al.*, 2008 ▸; Heaney *et al.*, 2001 ▸; Wirth, 2004 ▸; Meng *et al.*, 2011 ▸; Orloff *et al.*, 2003 ▸; Zhou & Wang, 2007 ▸; Yao, 2007 ▸). Okamoto *et al.* (2014 ▸) and Corbey *et al.* (2019 ▸) have already shown that using a FIB to prepare samples has advantages in the field of SC-XRD. Besides the preparation of small and well defined samples (Okamoto *et al.*, 2014 ▸), this preparation method can be used for especially challenging samples (Corbey *et al.*, 2019 ▸), such as (1) inclusions, (2) samples which are mixed with or embedded in undesirable materials, (3) materials which are difficult to grow as single crystals, and (4) hazardous materials that are, for example, radioactive or toxic. Corbey *et al.* (2019 ▸) compared results of the structure determination of well defined radioactive samples with different volumes. However, they neither discussed the influence of ion-beam radiation damage on the structure analysis nor compared different preparation techniques.

In this work, we used the pyroelectric material lithium niobate (LiNbO_3_) as reference system to investigate the influence of different crystal preparations on refined structure parameters and reconstructed EDs. The absorption of X-rays passing through a 50 µm-thick LiNbO_3_ crystal can reduce the transmission of Mo *K*α radiation by several tens of percent, which makes an absorption correction of the reflection intensities necessary. We aim to obtain improved atomic coordinates and reconstructed EDs for calculating polarizations and pyroelectric coefficients (to be published elsewhere). For compounds including atoms of small (*e.g.* lithium) and high (*e.g.* niobium) number of electrons *Z* in the same structure, such as in LiNbO_3_, this should be of high value. For our study we used already commercially available high-quality LiNbO_3_ crystals grown by the Czochralski method (Volk & Wöhlecke, 2008 ▸). Furthermore, the crystal structure of LiNbO_3_ is well known and has already been analyzed extensively (Abrahams, Reddy & Bernstein, 1966 ▸; Abrahams, Levinstein & Reddy 1966 ▸; Hamzaoui *et al.*, 2006 ▸; Hsu *et al.*, 1997 ▸; Etschmann & Ishizawa, 2001 ▸; Iyi *et al.*, 1992 ▸; Abrahams & Marsh, 1986 ▸).

This study utilized a FIB-based approach for the preparation of regularly shaped single crystals of a commercial standard material and evaluates the applicability of this approach for SC-XRD using a laboratory Bruker AXS diffractometer. We compare the refined structure parameters and reconstructed EDs of three differently prepared crystals, of which two were prepared by FIB and one was a manually cut crystal. We discuss the results with respect to reference data from spherical crystals and synchrotron data that have already been published (Etschmann & Ishizawa, 2001 ▸; Abrahams & Marsh, 1986 ▸; Hsu *et al.*, 1997 ▸; Iyi *et al.*, 1992 ▸).

## 2. Sample preparation   

Two different procedures were utilized: (1) FIB preparation for obtaining cube-shaped crystals, where one crystal is fixed to a tungsten tip, and (2) conventional mechanical preparation using a scalpel (as cut). For all preparation methods the same LiNbO_3_ thin plate wafer [surface area of (5 × 5) mm, thickness of 0.2 mm, obtained from CrysTec GmbH] polished on both surfaces was used. Although it is usually applied for the preparation of transmission electron microscope lamellae with dimensions of (1 × 10 × 15) µm, a FIB micro-fabrication sequence has been adapted here to cut well defined crystal samples with much larger volumes. Attention was paid to minimizing milling time because milling of large volumes (i) already necessitates long milling times and (ii) consumes the liquid metal ion source and thus increases costs, and finally (iii) extensive ion milling can cause ion radiation damage in the sample.

**Figure 1:**
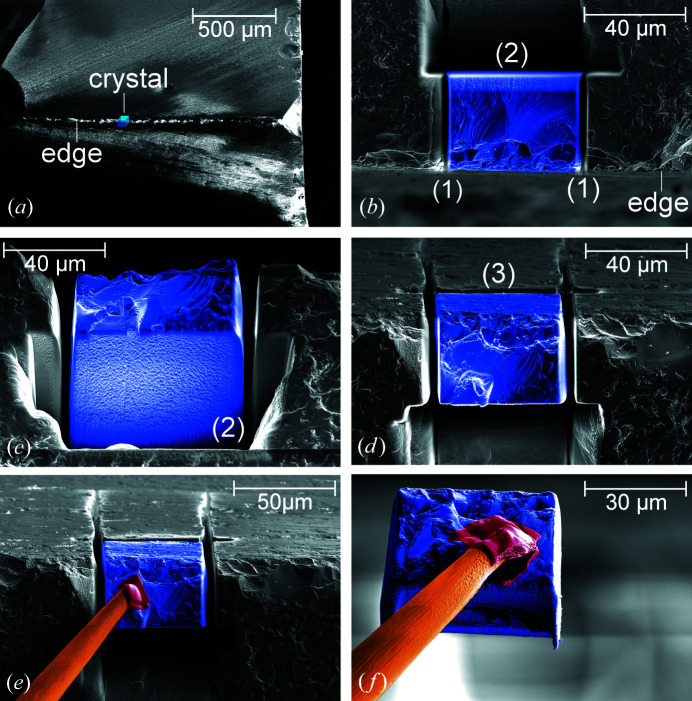
Crystal preparation using a FIB. The prospective crystal for SC-XRD is marked in blue. (*a*) outlines the cube-shaped crystal which will be cut out from the wafer edge. In the first preparation step (*b*) two trenches (1) perpendicular to the edge at a distance of 50 µm were cut, and then a staircase-like cross section parallel to the edge (2). For the final cutting step on the opposite side, the crystal was rotated by 180° around the electron-beam axis (*c*) and tilted as far as possible (−10°) in the reverse direction (*d*). In this position another trench (3) was cut. (*e*) shows the final crystal fixed on an easily transferable tungsten tip of the micro-manipulator (marked in orange). The platinum patch on the crystal surface (marked in red) links the crystal and tip so that the crystal can now be removed from the bulk *in situ* under microscope control (*f*).

The preparation was carried out in a dual-beam device (FEI Helios NanoLab 600i) equipped with a platinum gas injection system (Pt-GIS) and a Kleindiek micro-manipulator (MM3A) using a sharp tungsten tip. The gallium-ion-beam acceleration voltage was fixed at 30 kV, while the ion-beam current was adjusted between 9.3 and 21 nA depending on the size of the pattern area to achieve a constant current density. A freshly broken wafer, offering two already existing surfaces of the wanted cube, was utilized to reduce milling efforts [Fig. 1 ▸(*a*)].

**Figure 2:**
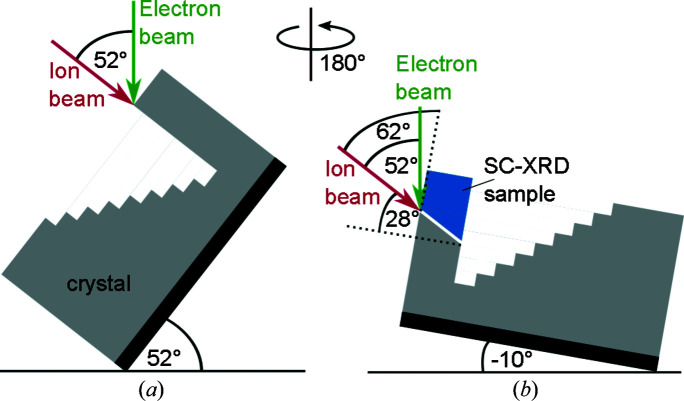
Scheme demonstrating the two different stage positions, *i.e.* the orientations and rotations of the crystal with respect to the electron and ion beams used for cutting (*a*) trenches (1) and (2) and (*b*) trench (3). For a stage tilt of 52° (rotation of 0°) the large crystal surface is perpendicular to the ion beam, while a stage tilt of −10° followed by a rotation of 180° results in an angle of 62° between the edge surface and the ion beam.

For the first two of three cuts [Figs. 1 ▸(*a*)–1 ▸(*d*)], the microscope stage was tilted by 52° in order to align the surface of the crystal perpendicular to the ion beam. After selection of the most suitable area at the wafer edge with perpendicular surfaces for the cube [Fig. 1 ▸(*a*)], two trenches were cut perpendicular to the edge with a depth of about 80 µm, slightly larger than the wanted edge length [Fig. 1 ▸(*b*)]. Then, a staircase-like cross section was cut to excavate the cube [Figs. 1 ▸(*b*) and 1 ▸(*c*)]. This step could be replaced by a trench cut but this would impede the later extraction of the cube. In the next step, the stage was tilted back across zero to the reverse direction as much as possible, in this case −10°, and then rotated by 180° (Fig. 2 ▸). The final cut was performed at the opposite side [indicated by (3) in Fig. 1 ▸(*d*)], so that the edge length of the sample was 50 µm. Afterwards, the crystal was still slightly connected to the bulk. Note that this last preparation step produces a miscut of 28° for one face of the crystal because the ion beam could not be aligned fully perpendicular to this particular crystal surface owing to limitations of the FIB system geometry (Fig. 2 ▸). Finally, a crystal possessing a well defined shape close to a cube was obtained [Fig. 1 ▸(*f*)].

**Figure 3:**
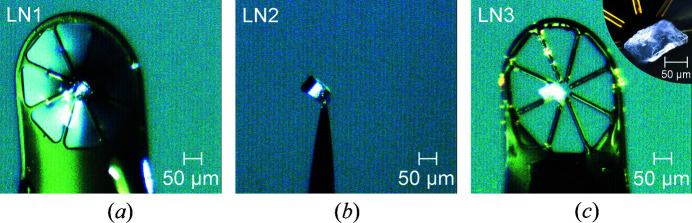
The three differently prepared crystals. (*a*) FIB-prepared cube-shaped crystal (LN1) mounted on a MicroGripper as crystal holder (olive), (*b*) FIB-prepared cube-shaped crystal (LN2) mounted on a tungsten micro-manipulator tip and (*c*) randomly shaped as-cut crystal (LN3) manually prepared from the same bulk material, also mounted on a MicroGripper. The inset in (*c*) shows a detailed view of the surface and shape of crystal LN3.

Two different procedures for fixing the FIB-prepared crystals to a sample holder were compared. The first crystal cube (LN1) was removed *ex situ* from the crystal plate under a laboratory microscope and fixed to a MiTeGen MicroGripper that was used as crystal holder [Fig. 3 ▸(*a*)]. The MicroGripper was then mounted onto a goniometer head and transferred to the diffractometer. After the second crystal cube (LN2) had been cut, a micro-manipulator tip was welded to the crystal *in situ* by platinum deposition [Fig. 3 ▸(*b*)]. The resulting platinum patch had a thickness of about 1–2 µm [Figs. 1 ▸(*e*) and 1 ▸(*f*)]. Once the FIB sample chamber had been opened, crystal LN2 was handled by just mounting the micro-manipulator tip onto a goniometer head that was then transferred to the diffractometer.

The FIB preparation was performed within approximately 6 h. To save preparation time, the use of wide trench milling instead of cross section milling reduces the time to 3 h but complicates removal of the crystal from the bulk. The rough surface of the original wafer edge (see Fig. 1 ▸), which represents one side of the crystal and originates from breaking of the wafer, induces thickness variations of less than 5 µm and thus absorption variations of less than 2%. This may be avoided by ion-beam treatment as well but would require an additional handling with longer preparation times and thus higher costs because of the extensive use of the liquid metal ion source.

**Figure 4:**
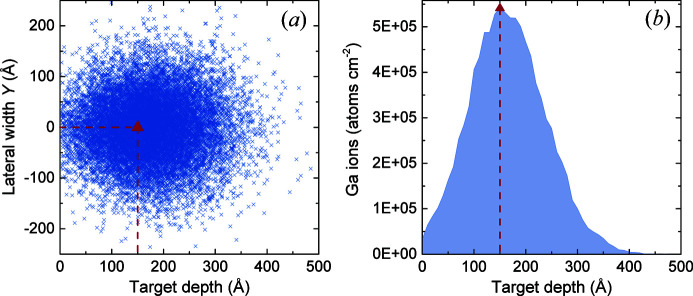
Near-surface distribution of implanted gallium ions induced by FIB milling into an LiNbO_3_ crystal calculated with SRIM (Ziegler *et al.*, 2010 ▸) (acceleration voltage: 30 kV). (*a*) shows the lateral and depth gallium distribution with maximum penetration levels of <250 Å and <500 Å, respectively, according to an alignment of the ion beam perpendicular to the crystal surface. (*b*) represents the number of gallium ions distributed along the crystal depth. The red triangles mark the positions of the highest density of gallium ions.

Stopping and range of ions in matter (SRIM) calculations (Ziegler *et al.*, 2010 ▸) perpendicular to the crystal surface have been performed according to the experimental parameters. The results are shown in Fig. 4 ▸. They indicate that the near-surface gallium-ion distribution for perpendicular irradiation is 480 Å at maximum, whereby the mean penetration depth is about 150 Å. In the lateral direction, the maximum penetration depth is 240 Å. Thus, the penetration of gallium ions in the LiNbO_3_ crystal is suggested to be not critical owing to the small irradiation damage volume. However, the calculation does not take the crystal structure or crystallographic characteristics into account. Energy-dispersive X-ray (EDX) analyses (see supporting information) confirm contamination of the crystal surface volume by gallium ions of the order of <21 at.%. Specific morphological changes could not be observed by scanning electron microscopy (SEM).

Note that changing between the ion beam for sample preparation and electron beam for SEM (for controlling the preparation process) charges the sample electrically, which changes the targeted crystal’s ion-beam position. This, in turn, leads to enlarged trenches and rounded edges. Therefore, control of the sample preparation by SEM should be kept to a minimum.

Finally, a third crystal (LN3) was prepared by manually breaking a small part from the wafer with a scalpel. Again, the MicroGripper was used as crystal holder [Fig. 3 ▸(*c*)]. The inset in Fig. 3 ▸(*c*) shows an optical micrograph for a detailed view of the crystal, which has approximate dimensions of (40 × 62 × 100) µm and an elongated shape, many concave facets and an overall rough surface. A schematic of the crystal is shown in  Fig. 3 in the supporting information.

## 3. Single-crystal X-ray diffraction   

**Figure 5:**
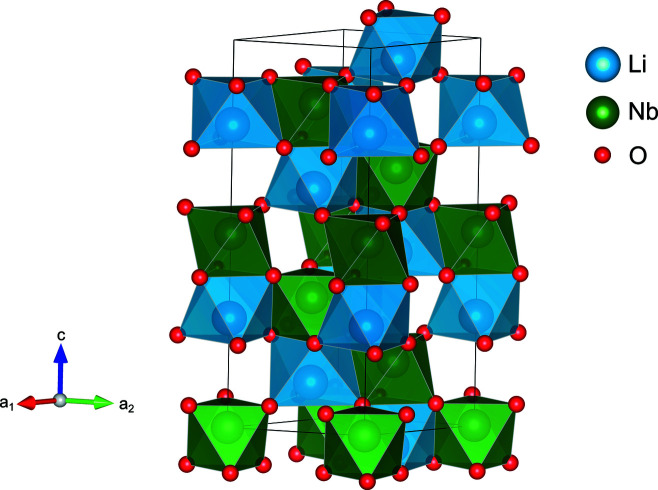
Crystal structure of LiNbO_3_ with space-group symmetry *R*3*c* (161).

The three prepared crystals were analyzed with an identical measurement strategy. For the diffraction experiment a Bruker D8 Quest (Bruker, 2012*b* ▸) single-crystal diffractometer with Mo *K*α radiation (wavelength λ = 0.71076 Å), an acceleration voltage of 50 kV and a tube current of 30 mA was used. The diffractometer was equipped with a Triumph monochromator and a Photon 100 area detector. The exposure time for a frame scan angle of 0.5° was set to 100 s. The measurements were performed at room temperature (296 K). Subsequently, the reflection intensities were corrected for absorption with a numerical approach based on the measured indexed crystal faces. We used the *APEX2* software (Bruker, 2012*a* ▸) for absorption correction and additionally verified the output with the program *X-SHAPE* (Stoe & Cie, 2002 ▸). Extinction correction (type 1, Lorentz mosaic distribution) and structure refinement against structure factors *F* were carried out using the program *JANA2006* (Petricek *et al.*, 2014 ▸). Finally, the ED was reconstructed separately with the maximum entropy method (MEM) using the program *BayMEM* (van Smaalen *et al.*, 2003 ▸). Here, we used a 36 × 36 × 72 voxel grid, the Sakata–Sato (Sakata & Sato, 1990 ▸) algorithm, static weighting (de Vries *et al.*, 1996 ▸) (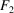) and the generalized *F* constraint (van Smaalen *et al.*, 2003 ▸) with order 2. From the ED, the atomic charges were determined with the program *EDMA* (Palatinus *et al.*, 2012 ▸) using the Bader charge formalism (Bader, 1990 ▸). The final refined structure is shown in Fig. 5 ▸. The experimental parameters of the different SC-XRD measurements and subsequent absorption corrections are listed in Table 1 ▸.

## 4. Influence of the preparation method on the recorded data and refined structure parameters   

As can be seen from Table 1 ▸, all prepared crystals exhibit an excellent data quality (*e.g.* small 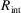 and 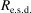). The redundancy, the number of measured reflections and the reflection intensities are high, as necessary for high-quality structure refinements. The number of measured reflections is more than 35% higher for crystal LN1 than for LN2 and LN3. Crystal LN1 also shows the highest data quality: the 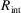 value is lower by 1.45 and 0.17% in comparison to crystals LN2 and LN3, respectively, while 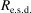 is only slightly increased because more low-intensity reflections were collected. For crystals LN2 and LN3 a smaller number of observed and total reflections exist. This is most likely due to the tungsten tip and platinum patch on the LN2 crystal surface [Figs. 1 ▸(*e*) and 1 ▸(*f*)] as well as to the fact that the LN3 crystal is 1.4 times larger (Table 1 ▸). Both factors induce a higher absorption of the X-ray radiation (*cf.* Table 1 ▸; 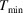 of LN2 and LN3 are the lowest). For an *x* = 1 µm-thick platinum layer a transmission *T* = 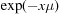 = 0.4 can be calculated using the platinum absorption coefficient μ = 931 mm^−1^ (Prince, 2004 ▸). Thus, this thin platinum layer transmits approximately half as much radiation as the full LiNbO_3_ crystal LN2 (*T*  =  0.8 with *x* = 50 µm and μ from Table 1 ▸). In other words, the platinum patch is responsible for an additional absorption of 60%, which is reflected in the lower intensity 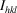 of selected reflections as well as by the lower number of recorded reflections (Table 1 ▸). In order to correct for the additional absorption due to the tungsten tip and platinum patch, the absorption correction algorithm increased the effective crystal volume by increasing the crystal thickness in the direction of the platinum patch by approximately 50%.

Table 2 ▸ compares the results of the structure refinements of the three crystals. Overall, all three crystals exhibit similar results with rather good quality parameters (low *R* values). The determined lattice parameters, atomic positions and equivalent displacement parameters (the full set of anisotropic displacement parameters is listed in the supporting information, Table 2) are equal within three times the standard deviation (3σ). Furthermore, the refinements resulted in rather low residual EDs.

Crystal LN2 exhibits significantly higher standard deviations for the atomic positions as well as larger *R* values than crystals LN1 and LN3. Crystal LN1 has slightly better 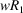 and 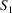 values than the other crystals. The quality of the refinement also needs to be rated in regard to the significantly larger number of reflections used for the refinement of crystal LN1, which can increase the absolute *R* value.

The 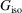 value describes the angular distribution of domains in the crystal and is given as the width of the distribution function of the extinction model used (Petricek *et al.*, 2014 ▸). A smaller 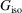 value means lower angular variation of domains and the extinction is independent of the domain size. Within 3σ, crystals LN1 and LN2, in comparison to LN3, have the smallest 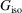 values. Note that the correction of the additional absorption influences affecting crystal LN2 (see above) might be partially compensated for by a slightly different extinction correction. Taking the total extinction correction into account, it can be seen that the low-indexed strong-intensity reflections at low scattering angles are differently affected for the three crystals. For crystal LN1, the extinction correction is the smallest (<6%), whereas for LN3 it is the highest (up to 18.9%). Thus, the FIB preparation technique seems to minimize extinction significantly and reproducibly.

The 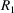 values and atomic coordinates obtained from the MEM-ED reconstructions (for more information see supporting information, Table 3) are comparable to those obtained by structure refinement (Table 2 ▸) and follow a similar trend.

**Figure 6:**
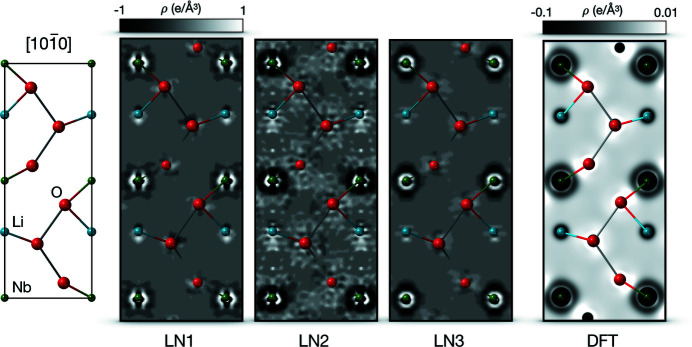
Sections of difference EDs (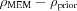; aspherical contributions) perpendicular to the [10
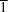
0] direction for crystals LN1, LN2 and LN3. A schematic view of the section is provided on the left, while the difference EDs of the different crystals are shown in the center. The experimental ED is most blurred for the LN2 crystal. For comparison purposes, a theoretical difference ED calculated from a spherical IAM-ED and an aspherical DFT-ED is shown on the far right. The theoretical ED does not include thermal smearing.

Difference ED maps 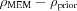 were calculated on the basis of the MEM-ED 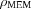 and a ‘prior’ ED 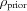 in order to further evaluate the FIB preparation. 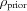 is a reference ED used for MEM calculation (Palatinus & van Smaalen, 2002 ▸) and estimated with the independent atom model (IAM) (Coppens, 1997 ▸). 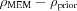 maps can be interpreted in terms of the deformation of the ED due to chemical bonding (van Smaalen *et al.*, 2003 ▸). Fig. 6 ▸ shows the complete unit cell in the [10
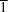
0] direction and corresponding difference EDs. Additionally, a theoretical difference ED calculated from a spherical IAM-ED and an aspherical DFT-ED (calculated at 0 K) is shown for comparison purposes. For the density functional theory (DFT) calculation a Γ-centered 12 × 12 × 4 Monkhorst–Pack (Monkhorst & Pack, 1976 ▸) *k*-grid with an energy cut-off of 600 eV for the plane-wave basis set and PBE-PAW (Kresse & Joubert, 1999 ▸; Perdew *et al.*, 1996 ▸) as implemented in *VASP* (Kresse & Furthmüller, 1996 ▸) were used. The aspherical density represents the fully converged ground state.

Comparing the difference EDs, all crystals show similar features, although the experimental ED for crystal LN2 is noisier. In contrast, the ED of crystal LN1 is least noisy and compares, together with that of crystal LN3, well with the theoretical ED. A more detailed discussion of the ED maps can be found in the supporting information.

## 5. Discussion   

We successfully prepared single crystals for SC-XRD utilizing a FIB technique. Crystals with a reproducible and defined shape and size were obtained. Corbey *et al.* (2019 ▸) reported a similar study for the FIB preparation of radioactive materials intended for a crystal structure analysis. In comparison to Corbey *et al.* (2019 ▸), we performed smaller cuts with the ion beam to reduce beam damage on the sample. We also compared the results of the structure refinement of FIB-prepared crystals with those of crystals prepared via commonly used techniques as well as with literature data obtained from, for example, ground spheres or as-grown crystals (Table 3 ▸), for a better evaluation of the influence of the FIB preparation on the structure refinement. Note that the data were recorded with a D8 Quest Bruker AXS diffractometer and corrected with the corresponding *APEX2* software; other software or diffractometers might influence the absorption correction or the results differently.

The collected diffraction data, determined lattice parameters, structure refinement characteristics, and corresponding atomic positions and displacement parameters as well as the MEM-reconstructed EDs are of high quality but show only small differences for all crystals. Even when a micro-manipulator tip is fixed onto a crystal surface (crystal LN2), the structure refinement is still of high quality and the structure parameters are comparable; however, the difference ED is more blurred and noisy. The absolute *R* values are still in the range of high-quality data (Massa, 2011 ▸). The differences in refinement quality parameters between the FIB and manually prepared crystals are rather small; the improvements of the *R* values (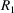 and 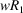) are smaller than 0.1%. The same applies to the standard deviations.

Sample LN2 shows slightly higher deviations, which are predominantly induced by the additional platinum patch and tungsten tip, making the absorption correction difficult to evaluate. Here, we estimated the additional absorption influences with the increase of the effective crystal volume. This is just an approximation and further work on modeling of the absorption profile for regions with different composition and absorption influence in one sample is encouraged. The disadvantages concerning the preparation of sample LN2 could be decreased by changing the tip and patch material, which needs further investigation as well. However, the findings concerning crystal LN2 are important given that this approach allows a simpler handling of crystals from complex samples that can be directly removed and transferred to the diffractometer. Furthermore, the time of direct contact with the sample could be reduced to a minimum, which is important especially for hazardous samples (Corbey *et al.*, 2019 ▸). Corbey *et al.* (2019 ▸) presented an additional procedure for transferring the samples from the FIB tip to a MiTeGen MicroMount and fixing that sample with a carbon patch. Although this is an extra preparation step, the influence of additionally absorbing materials can be minimized.

Apart from crystal LN2, there seems to be no significant difference in the refined structure parameters and difference EDs. However, the extinction influences are significantly different. We interpret the findings as follows: The ion beam destroys the crystallinity of the crystal’s surface (LN1) in the near-surface region and forms an amorphous inhomogeneous layer (Rubanov & Munroe, 2004 ▸). According to the SRIM simulations (Fig. 4 ▸) the layer thickness is approximately 0.024 µm. The ratio of the surface layer volume to the crystal’s volume then amounts to 0.3%. This results in a significantly (>9%) reduced extinction effect. As Boehm *et al.* (1974 ▸) already reported, such surface damage has an influence on the reflection intensities, but a detailed and quantitative interpretation of the results with respect to the extinction was not given. The theoretical description of the extinction [see *e.g.* Becker & Coppens (1974 ▸) (used in *JANA2006*), Hamilton (1957 ▸) or Zachariasen (1967 ▸)] assumed homogeneity through­out the crystal. Theories for extinction need to be modified to include a model of a relatively perfect crystal enclosed by an imperfect amorphous layer. Such a theory would require additional parameters describing the dimensions and states of perfection of the homogeneous crystal core and the inhomogeneous volume near the surface (Boehm *et al.*, 1974 ▸) and is thus sufficiently complex to be beyond our purpose.

An exact extinction correction, however, is important for high-quality EDs and the reduction of systematic errors of the recorded reflection intensities. Especially for strong reflections at low scattering angles 2θ the impact of extinction is strong. An inaccurate absorption correction due to an irregularly shaped crystal can also introduce errors, which are then compensated for, in part, by the extinction correction during refinement.

Furthermore, the preceding absorption correction could be affected by the amorphous layer and gallium implantation (detected by EDX, see supporting information, Table 1). As already indicated above, the absorption correction assumes a uniform absorption cross section through the crystal and does not include separate absorption factors (Boehm *et al.*, 1974 ▸; Lee & Ruble, 1977 ▸). This condition of uniformity is in fact not fully fulfilled here and deserves an extended model of the absorption of non-uniform crystals. To the best of our knowledge the extinction and absorption correction of inhomogeneous samples is not implemented in commonly used software for structure determination and refinement of SC-XRD data (*e.g.* Farrugia, 2012 ▸; Spek, 2009 ▸; Sheldrick, 2015 ▸; Petricek *et al.*, 2014 ▸; Bruker, 2001 ▸).

The gallium-ion-beam treatment has a negligible influence on the average crystal structure as well as on the structure refinement owing to the comparatively low penetration depth of the gallium ions (Fig. 4 ▸) and thus the small affected volume fraction of approximately 0.3%. Unwanted structural changes caused by enhanced lithium diffusion due to localized heating and/or by the electrostatic charging induced by the electron beam, as was reported by Wang & Meng (2016 ▸), could not be detected here. The same applies to the already known recrystallization due to the generated heat upon gallium-ion irradiation from transmission electron microscopy (TEM) sample preparation (Rubanov & Munroe, 2004 ▸; Kato, 2004 ▸). Other drawbacks of FIB preparation are increased surface roughness, thickness non-uniformities, induced atomic defects, implantation of gallium and sample alterations due to beam heating in general (Kato, 2004 ▸). Thus, a detailed clarification of how FIB alteration of the crystal structure affects the extinction and absorption correction for SC-XRD is of interest. This also includes checking the applicability of processes for alteration reduction from the literature. Such processes are dry or wet etching and ‘cleaning’ of the sample with a low-energy FIB or with conventional broad argon-ion milling subsequent to fabrication (Kato, 2004 ▸). This is already established for TEM sample preparation but further increases the preparation efforts. Nevertheless, as discussed above, for SC-XRD we see the most significant influence of the FIB preparation to be on the extinction.

The determined (difference) EDs of all crystals are comparable to the theoretically calculated ED but are more blurred, which is attributed to the thermally induced blurring of the ED at room temperature. We rate the charge values of crystal LN1 (Table 2 ▸) as more reliable, especially in regard to the low-scattering lithium ions; the experimental value resembles the theoretical one (+0.9 electrons; see supporting information, Table 5). Since the lithium–oxygen bond is characterized as an ionic bond, the partitioning algorithm generates more unambiguous charge values. In contrast, for the more covalently bonded niobium–oxygen bond the determined charges deviate strongly. Here, owing to the overlap of the EDs of the atoms – and the thermal smearing – the partitioning cannot clearly allocate the charges. This might explain the slightly lower experimental charges for the niobium (theoretical value 3.1 electrons) and oxygen (theoretical value −1.3 electrons) ions.

For high-precision SC-XRD measurements, for example ED or charge-density determination, usually spherical crystals are used. In order to further evaluate the quality of the FIB-prepared crystal, we compare our results with high-quality data obtained from spherical and small as-grown crystals. Table 3 ▸ provides literature data on *R* values and further experimental details.

Regarding Table 3 ▸, the data quality of our FIB-prepared crystal measured at a laboratory source is comparable to and of the same high quality as the synchrotron data. Note that refinement on *F* usually generates significantly lower *R* values in comparison to refinement on 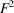 (*e.g.* Abrahams & Marsh, 1986 ▸). Furthermore, our reconstructed difference ED is comparable to those reported by Etschmann & Ishizawa (2001 ▸) and Hsu *et al.* (1997 ▸), although our data are characterized by fewer artifacts and less blurring. This could, however, mainly be due to the applied MEM approach, which better estimates non-measured reflections (structure factors) with their statistically most likely values (Coppens, 1997 ▸).

Overall, we rate the structure parameters and calculated charges, *i.e.* oxidation numbers, more reliable for crystal LN1 since the absorption correction is less ambiguous and the extinction influence is significantly reduced (Table  2 ▸).

## 6. Conclusion   

Here we presented a study on an alternative preparation method for SC-XRD samples, applying a FIB technique to obtain crystals with a defined size and shape reproducibly. The experimental data and data refinement quality in terms of the rating *R* values are in excellent agreement with an as-cut crystal as well as with literature data based on spherical crystals or synchrotron radiation. In contrast to the anticipated improvement of the structure refinement, especially when light and heavy atoms are present in the same structure, we observed no significant influence on the crystal structure parameters except for a significant reduction of the extinction. However, we suggest that the absolute values of structure parameters and ED are of higher quality.

No drawbacks of the gallium-ion milling and corresponding alteration of the crystal structure on the refined structure parameters were identified. Even fixing a tungsten tip with a platinum patch on a FIB-prepared crystal, which faciliates handling of crystals, still leads to data sets and refined structure parameters of comparatively good quality. Changing the material of the tip and patch to low-*Z* materials could minimize additional systematic absorption influences.

We encourage further investigations concerning the influence of radiation damage due to the ion-beam milling process during FIB preparation. This applies, for example, to an amorphous surface layer, gallium implantation, and improvements for extinction and absorption correction approaches which take crystal inhomogeneities into account. Here, the already existing knowledge from the TEM community concerning the influence of radiation damage upon FIB preparation and subsequent treatments for removal of damage could be of high value.

In conclusion, we highly recommend the presented minimally invasive preparation method for application in the field of crystallography since no significant drawbacks were detected. Furthermore, the reduction of extinction and ambiguities of the crystal shape determination for absorption correction was observed. However, the positive effect of FIB preparation is less pronounced than expected, and the method is more time consuming and costly than conventional techniques. Thus, we recommend a cost–benefit estimation in advance of the experiment.

Therefore, this method should only be used in cases when a specific area of a macroscopic sample (*e.g.* domains, inclusions) or a complex material architecture (*e.g.* a doped crystal or semiconductor element) will be investigated, when the crystal shape needs to be defined (*e.g.* property measurements), or when reliable EDs are sought. The FIB preparation method might also be useful for susceptible samples, *e.g.* brittle, soft, thin, hard, hazardous or toxic materials.

## 7. Related literature   

The following additional literature is cited in the supporting information: Henke *et al.* (1993 ▸).

## Supplementary Material

Crystal structure: contains datablock(s) global, LN1, LN2, LN3. DOI: 10.1107/S1600576720003143/in5030sup1.cif

Structure factors: contains datablock(s) global, I. DOI: 10.1107/S1600576720003143/in5030LN1sup2.hkl

Structure factors: contains datablock(s) global, I. DOI: 10.1107/S1600576720003143/in5030LN2sup3.hkl

Structure factors: contains datablock(s) global, I. DOI: 10.1107/S1600576720003143/in5030LN3sup4.hkl

Additional information and data on the sample preparation and structure refinement. DOI: 10.1107/S1600576720003143/in5030sup5.pdf

CCDC references: 1988683, 1988684, 1988685

## Figures and Tables

**Table 1:** Experimental parameters of the differently prepared LiNbO_3_ crystals ‘obs’ and ‘all’ correspond to observed [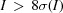] and all recorded reflections with intensity *I*; 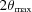 is the maximum diffraction angle, *h*, *k*, *l* are the Miller indices, *R* corresponds to the different quality parameters of the data sets {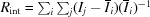 considers the integrated reflection intensities while 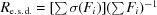 considers the estimated standard deviation (e.s.d.) values of the structure factors; Petricek *et al.*, 2014 ▸}, μ is the linear absorption coefficient, *T* is the transmission of X-rays and 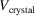 is the volume of the investigated crystals according to the absorption correction.

Crystal	LN1	LN2	LN3
Preparation method	FIB	FIB + tip	As cut
Crystal shape	Cubic	Cubic	Random
Approximate crystal size (µm)	50 × 50 × 63	50 × 50 × 63	40 × 62 × 100

2θ_max_ (°)	117.4		
λ(Mo *K*α) (Å)	0.71076		
sinθ_max_/λ (Å^−1^)	1.2		
*h*, *k*, *l*	−12 < *h* < 12		
	−12 < *k* < 9		
	−32 < *l* < 32		

Reflections (obs/all)	10271/11592	7644/8594	7422/8389
Redundancy	10.0	7.4	7.2
*R*_int_ (obs/all) (%)	2.93/2.94	4.38/4.38	3.10/3.10
*R*_e.s.d._ (obs/all) (%)	0.44/0.46	0.30/0.30	0.35/0.36
μ (mm^−1^)	5.3270	5.3270	5.3270
*T*_min_	0.76	0.57	0.67
*T*_max_	0.82	0.76	0.85
*V*_crystal_ (10^−4^ mm^3^)	1.51	2.07	2.14

Reflection intensity *I* *_hkl_* (a.u.)			
102	768 352	152 500	113 650
006	279 577	92 905	40 847
110	435 106	133 506	85 186

**Table 2:** Refinement and structure parameters of the differently prepared LiNbO_3_ crystals ‘obs’ and ‘all’ correspond to observed [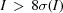] and all recorded reflections, *Z* is the formula unit, *M* the molar mass, ρ_X-ray_ the ‘ideal’ mass density, *a* and *c* the lattice parameters, 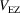 the unit-cell volume, 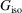 the extinction parameter (Petricek *et al.*, 2014 ▸), *x*, *y*, *z* the fractional atomic coordinates, 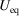 the equivalent displacement parameter (Petricek *et al.*, 2014 ▸), and 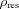 the residual ED. The refinement was based on *F* with the goodness-of-fit parameter 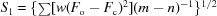 and the quality-of-refinement values 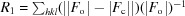 and 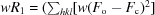
 × 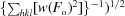. Below, the results of the MEM-ED reconstruction and corresponding Bader charges are summarized. All the given errors correspond to 1σ.

Crystal	LN1	LN2	LN3
*Z*	6	6	6
*M* (g mol^−1^)	147.84	147.84	147.84
ρ_X-ray_ (g cm^−1^)	4.62	4.62	4.62
Bravais lattice	Rhombohedral (hexagonal setting)		
Space group	*R*3*c* (161)	*R*3*c* (161)	*R*3*c* (161)
*a* (Å)	5.1505 (1)	5.1513 (1)	5.1516 (1)
*c* (Å)	13.8683 (4)	13.8687 (6)	13.8690 (6)
*V*_EZ_ (Å^3^)	318.74 (1)	318.71 (2)	318.76 (2)
*F*_000_	408	408	408
Extinction correction	Isotropic, type 1, Lorentz (Petricek *et al.*, 2014 ▸)		
*G*_iso_	0.05 (1)	0.14 (2)	0.26 (1)

Extinction influence on reflection *hkl* (%)			
102	5.8	9.2	18.9
006	1.3	4.0	7.8
2 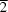 16	0.2	0.7	1.2

Li atom			
*x*	0	0	0
*y*	0	0	0
*z*	0.2803 (4)	0.2804 (9)	0.2815 (4)
*U*_eq_ (Å^2^)	0.0087 (8)	0.0094 (15)	0.0082 (8)

Nb atom			
*x*	0	0	0
*y*	0	0	0
*z*	0.000057 (4)	0.000045 (7)	0.000055 (3)
*U*_eq_ (Å^2^)	0.00497 (2)	0.00556 (4)	0.00493 (2)

O atom			
*x*	0.0479 (1)	0.0480 (2)	0.0489 (1)
*y*	0.3429 (1)	0.3426 (2)	0.3438 (1)
*z*	0.06377 (8)	0.06390 (14)	0.06342 (8)
*U*_eq_ (Å^2^)	0.0072 (1)	0.0079 (2)	0.0073 (1)

ρ_res_ (e Å^−3^)	1.17/ 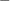 0.92	1.21/ 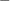 1.82	0.79/ 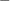 1.06
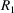 (obs/all) (%)	1.63/1.82	2.87/2.94	1.52/1.63
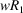 (obs/all) (%)	2.04/2.07	3.82/3.83	2.07/2.09
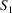 (obs/all)	1.62/1.54	3.18/3.09	1.69/1.65

MEM-ED reconstruction			
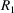 (%)	1.71	2.74	1.59
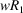 (%)	2.04	3.60	2.03

Bader charges			
Li (e)	0.9	0.7	0.7
Nb (e)	1.6	1.9	2.4
O (e)	−0.7	−0.9	−1.0

**Table 3:** Literature data of high-quality SC-XRD of differently prepared LiNbO_3_ single crystals Note that all refinements were based on *F*, except that by Abrahams & Marsh (1986 ▸) who refined on *F* ^2^.

	Etschmann & Ishizawa (2001 ▸)	Abrahams & Marsh (1986 ▸)	Hsu *et al.* (1997 ▸)	Iyi *et al.* (1992 ▸)
Source	Synchrotron	X-ray tube	Synchrotron	X-ray tube
	Wiggler		Wiggler	
Crystal shape	Spherical	Spherical	Rectangular	Spherical
Crystal size (µm)	160†	180†	140 × 50 × 77	140†
sinθ_max_/λ (Å^−1^)	1.21	1.1	1.1	1.21
Reflections (all)	6197	4011	4622	948 (unique)
Refinement on	*F*	*F* ^2^	*F*	*F*
*R*_int_ (%)	2.4	0.96	3.99	0.777
*R* (%)	1.04	1.23	1.5	1.12
*wR* (%)	1.03	1.63	1.5	1.38
*S*	1.99	2.06	3.28	1.56

† Diameter.
