# Supplementary material for: X-ray diffraction using focused-ion-beam-prepared single crystals
Source: J Appl Crystallogr. 2020 Apr 14;53(Pt 3):614–22. doi: 10.1107/S1600576720003143 (PMC7312134; doi:10.1107/S1600576720003143)
Supplement: Supplementary file 5 [file j-53-00614-sup5.pdf]

Supplementary information

Tina Weigel,[a] Claudia Funke,[a] Matthias Zschornak,[a] Thomas Behm,[a] Hartmut Stöcker,[a] Tilmann Leisegang[a,b] and Dirk C. Meyer[a]

[a]*Institute of Experimental Physics, Technische Universität Bergakademie Freiberg, Leipziger Str. 23, 09596 Freiberg, Germany, and* [b]*Samara State Technical University, Molodogvardeyskaya Street 224, 443100 Samara, Russia*

1. Transmission of X-ray radiation in $LiNbO_3$

The calculation of transmission of X-ray radiation through 50 µm $LiNbO_3$ for an energy range of (0–30) keV is shown in Fig. 1. This was calculated with the Henke *et al.* (1993) database.

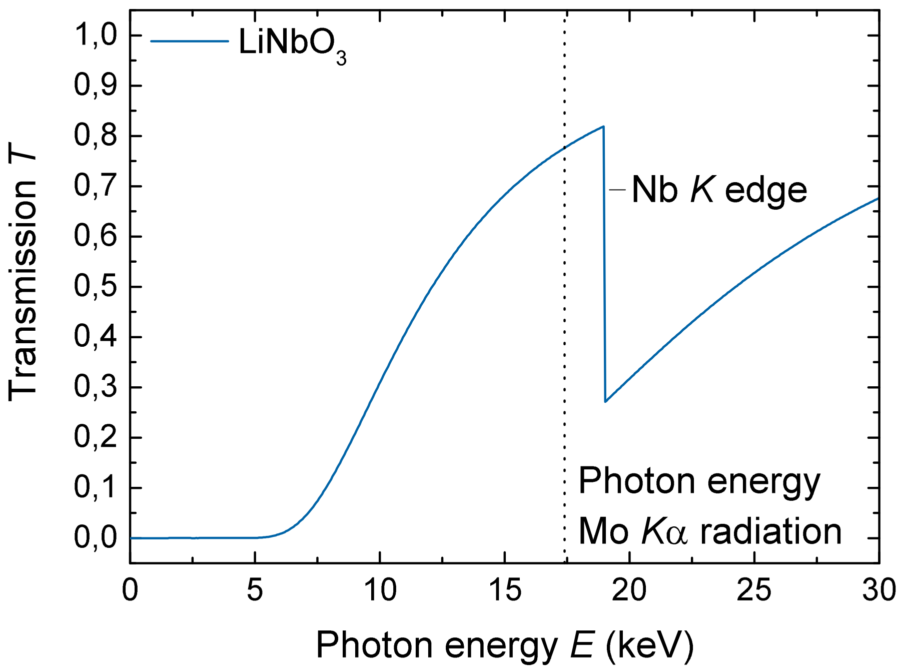

Fig. 1. Transmission of X-ray radiation through 50 µm thick $LiNbO_3$. The used photon energy of Mo $K\alpha$ is marked with a dotted line. The Nb K edge ($E_{\mathrm{Nb}K} = 18.986$ keV) is indicated (Henke *et al.*, 1993).

The absorption of X-rays passing through a 50 µm thick $LiNbO_3$ crystal can reduce the transmission of Mo $K\alpha$ radiation by approximately 20%. This makes an absorption correction of the reflection intensities necessary. Due to the gallium-ion treatment of the sample, a significant contamination with gallium occurs near the cut trenches. To quantify the gallium contamination, we analyzed the gallium concentration by energy dispersive X-ray (EDX) spectroscopy. The EDX data are listed in Tab. 1. Accordingly, a significant amount of gallium of 21(5) at.% is found in the near surface volume. An analysis of Li with EDX spectroscopy was not possible.An analysis of Li with EDX spectroscopy was not possible. However, if we suppose an expected value of 20 at.%, the amount of Ga would be ¡18 at.%.

Table 1. *Results of the EDX analysis.*

Element	Concentration (at.%)
Ga	21(5)
Nb	37(8)
O	42(8)

The samples LN1 and LN2 were prepared from a crystal plate with surface area of 5 x 5 mm^2, thickness of 0.2 mm, obtained from CrysTec GmbH and polished on both surfaces (see Fig. 2). The prepared samples LN1 and LN2 had a regular, defined shape (see Fig. 3a) in comparison to the manually broken sample LN3 with an irregular, elongated shape with many concave facets (see Fig. 3b).

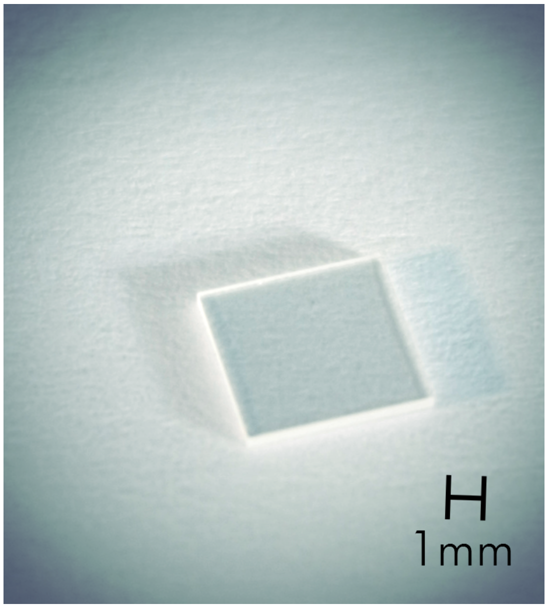

Fig. 2. Crystal plate, which was used for FIB-preparation.

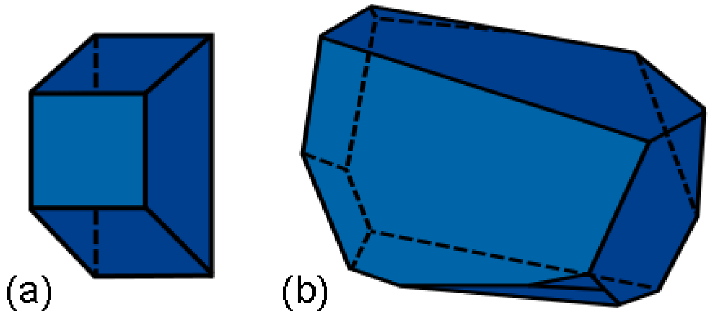

Fig. 3. Scheme illustration of the crystal shape from the FIB-prepared samples LN1 and LN2 (a) as well as from the manually broken sample LN3 (b). The illustration is not true to scale.

2. Anisotropic thermal displacement parameters

For better comparability, the equivalent thermal displacement parameters U_{eq} (Petricek *et al.*, 2014), calculated from the anisotropic thermal displacement parameters, are presented in Tab. 2 of the main document. U_{eq} reduces all the anisotropic thermal displacement parameters to one parameter. For a more detailed analysis, the anisotropic thermal displacement parameters U_{ij} are listed in Tab. 2.

Table 2. *Refined anisotropic thermal displacement parameters given in* Å^2.

Crystal	LN1	LN2	LN3
**Li atom**			
$U_{11} = U_{22}$	0.0073(8)	0.0070(15)	0.0069(8)
U_{33}	0.012(2)	0.014(3)	0.011(2)
U_{12}	0.0036(4)	0.0035(8)	0.0034(4)
$U_{13} = U_{23}$	0	0	0
**Nb atom**			
$U_{11} = U_{22}$	0.00528(3)	0.00576(5)	0.00520(3)
U_{33}	0.00434(3)	0.00518(6)	0.00441(3)
U_{12}	0.0026(1)	0.0029(3)	0.0026(1)
U_{13}/U_{23}	0	0	0
**O atom**			
U_{11}	0.0081(2)	0.0088(2)	0.0080(2)
U_{22}	0.0060(1)	0.0063(2)	0.0060(1)
U_{33}	0.0076(1)	0.0083(2)	0.0077(1)
U_{12}	0.0034(2)	0.0036(2)	0.0034(1)
U_{13}	−0.0013(2)	−0.0014(2)	−0.0014(1)
U_{23}	−0.0021(1)	−0.0022(2)	−0.0023(1)

All parameters are equal within three standard deviations (3σ), except the main

diagonal elements of Nb.

3. Detailed analysis of the difference ED

The electron density (ED) was reconstructed from experimental data with the maximum entropy method (MEM) and analyzed with the program EDMA to determine the atomic positions. The results are listed in Tab. 3.

Table 3. *Parameters of ED reconstruction with the MEM and corresponding atomic positions determined by EDMA and given in fractional coordinates. "coc" denotes the center-of-charge whereas the other given coordinates refer to the maximum ED.*

Crystal	LN1	LN2	LN3
Algorithm	SAKATA-SATO (Sakata & Sato, 1990)		
R_1 (%)	1.71	2.74	1.59
wR_1 (%)	2.04	3.60	2.03
Number of Voxels	36 x 36 x 72		
Prior density	non-uniform prior		
Static weighting	F_2 (De Vries *et al.*, 1996)		
Generalised F-constraint n	2 (van Smaalen *et al.*, 2003)		
z_{Li}	0.280079	0.279430	0.280093
z_{Nb}	−0.000036	0.000019	0.000036
x_{O}	0.045966	0.046332	0.045937
y_{O}	0.340802	0.338773	0.338229
z_{O}	0.064844	0.065776	0.065973
$z_{\mathrm{coc,\,Li}}$	0.279838	0.278974	0.277778
$z_{\mathrm{coc,\,Nb}}$	0.000000	0.000000	0.000000
$x_{\mathrm{coc,\,O}}$	0.048485	0.049584	0.048066
$y_{\mathrm{coc,\,O}}$	0.341576	0.344141	0.345744
$z_{\mathrm{coc,\,O}}$	0.063635	0.063719	0.066247

The atomic coordinates and R_1 values of MEM reconstructions and structure refinements (*cf.* Tab. 2 of the main document) are similar. Two main peculiarities exist. First, the coordinates of the center-of-charge for the niobium atom is 0, 0, 0 whereas the maximum ED is slightly shifted. Second, the z_{Nb} coordinate of crystal LN1 is now slightly negative. This, however, corresponds well with theoretical structure data from density functional theory (DFT) calculations (Tab. 4) and is another indication of the excellent diffraction data quality of crystal LN1. The aspherical density represents the fully converged ground state.

Table 4. *Details and structural parameters of aspheric DFT-ED for $LiNbO_3$.*

Parameter	DFT
Voxel	216 x 216 x 576
a (Å)	5.232061
c (Å)	14.029415
V_{EZ} ($\mathrm{Å}^3$)	332.410607
**Li atom**	
x	0
y	0
z	0.280570
**Nb atom**	
x	0
y	0
z	-0.00002
**O atom**	
x	0.044019
y	0.342021
z	0.075493

Difference ED maps $\rho_{\mathrm{MEM}} - \rho_{\mathrm{prior}}$ were calculated based on the MEM-ED ρ_{MEM} and a prior ED ρ_{prior} in order to further evaluate the FIB-preparation. The theoretical difference ED was calculated from a spherical IAM-ED and an aspherical DFT-ED (calculated at 0 K). Fig. 4 shows corresponding sections of the difference ED for the lithium–oxygen and niobium–oxygen plane perpendicular to the $[\bar{1}100]$ and $[1\bar{1}00]$ directions, respectively.

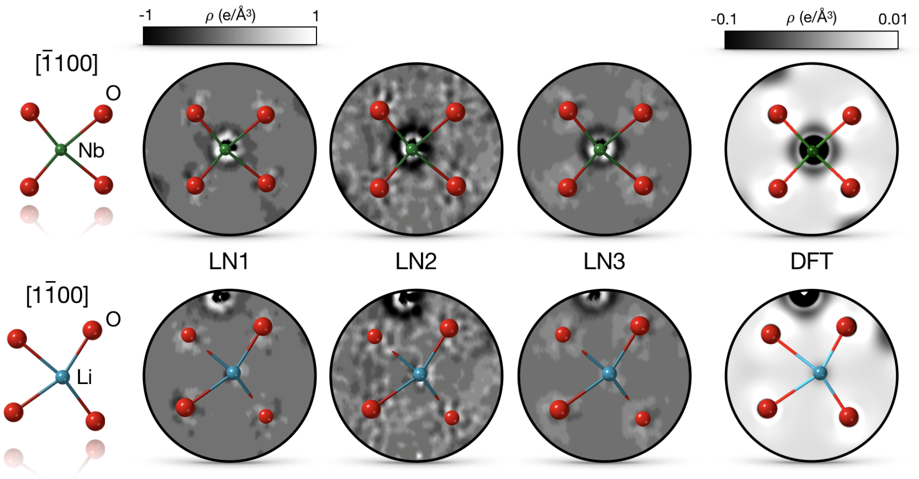

Fig. 4. The difference ED $\rho_{\mathrm{MEM}} - \rho_{\mathrm{prior}}$ for crystals LN1, LN2, and LN3 shows a detailed view of the niobium–oxygen plane perpendicular to [$\bar{1}$100] and the lithium–oxygen plane perpendicular to [1$\bar{1}$00]. For comparison purposes, a theoretical difference ED calculated from a spherical IAM-ED and an aspherical DFT-ED is additionally shown. It can be seen that the experimental ED is most blurred for the LN2 crystal. It has to be noted that the theoretical ED does not include thermal smearing.

The difference ED maps show a pronounced ED between niobium and oxygen. This indicates a covalent bond with a bond-critical point having $(0.017\ldots0.019)\,\mathrm{e\AA}^{-3}$ $(0.16\ldots0.19)\,\mathrm{e\AA}^{-3}$, $(0.54\ldots0.59)\,\mathrm{e\AA}^{-3}$, and $(0.17\ldots0.22)\,\mathrm{e\AA}^{-3}$ for the DFT data, crystal LN1, LN2, and LN3, respectively. Due to the distorted niobium-oxygen octahedron, the bond critical points vary for every niobium-oxygen bond. Again, thermal atomic vibrations should have an impact on the values and impede a comparison of experiment and theory. Lithium and oxygen bonds exhibit no overlapping ED (Fig. 4) which indicates an ionic bond.

Additionally, a Bader analysis (Bader, 1990) of the EDs was carried out to determine atomic charges and to calculate oxidation numbers. The results are summarized in Tab. 5.

Table 5. *Oxidation numbers calculated by subtracting the atomic number Z from the* BADER *charges.*

Crystal	LN1	LN2	LN3	DFT
Li charge (e)	0.9	0.7	0.7	0.9
Nb charge (e)	1.6	1.9	2.4	3.1
O charge (e)	−0.7	−0.9	−1.0	−1.3

The general trend of the oxidation numbers is comparable although the thermal vibrations have not been considered by DFT. Whereby the lithium value for crystal LN1 is in excellent agreement with DFT, however, the values for niobium and oxygen of crystal LN3 are closer to DFT. A further analysis will be presented elsewhere.

References

Bader, R. F. (1990). *Atoms in molecules.* Wiley Online Library.

De Vries, R. Y., Briels, W. J. & Feil, D. (1996). *Physical Review Letters*, **77**(9), 1719.

Henke, B. L., Gullikson, E. M. & Davis, J. C. (1993). *Atomic Data and Nuclear Data Tables*, **54**(2), 181–342.

Petricek, V., Dusek, M. & Palatinus, L. (2014). *Zeitschrift für Kristallografie*, **229**, 345–352.

Sakata, M. & Sato, M. (1990). *Acta Crystallographica Section A: Foundations of Crystallography*, **46**(4), 263–270.

van Smaalen, S., Palatinus, L. & Schneider, M. (2003). *Acta Crystallography A*, **59**, 459–469.
